# The Spread of Endogenous Retroviruses in Populations Infected by Exogenous Viruses

**DOI:** 10.3390/v17060770

**Published:** 2025-05-28

**Authors:** Hyunjin Park, Paul G. Higgs

**Affiliations:** Department of Physics and Astronomy, McMaster University, Hamilton, ON L8S 4L8, Canada

**Keywords:** retroviruses, endogenous viruses, maternal transmission, evolution of virulence

## Abstract

Retroviruses insert DNA copies of themselves into the chromosomes of their hosts forming proviruses that can synthesize new transmissible viruses. Exogenous retroviruses (XRVs) insert into the DNA of somatic cells and are transmitted infectiously. Endogenous retroviruses (ERVs) become inserted in the DNA of germline cells and are transmitted genetically. ERVs can spread through the genome by transposition. ERVs originate from an initial copy of an XRV inserted into the genome of an organism infected by the XRV. Many XRVs are transmitted maternally as well as horizontally; therefore, we consider the effect of maternal transmission on the evolution of virulence of an XRV. Our model shows that the XRV either evolves high virulence with low maternal transmission, or vice versa. We then consider the spread of ERV genes in conjunction with the infectious spread of an XRV. Beginning from a single copy of an ERV, we calculate the probability that it spreads to fixation (i.e., the state where all individuals contain ERV genes). This depends on its virulence and transposition rate. If the XRV is present, the fixation probability also depends on the virulence of the XRV and whether the ERV provides resistance to the XRV. An ERV with only a small deleterious effect on host fitness has a high fixation probability, particularly if it provides resistance to the XRV. We also show that, if an ERV does not spread to fixation, it can still cause elimination of the XRV, with the end result that the population is cleared of both XRV and ERV.

## 1. Introduction

Retroviruses are a unique class of RNA viruses that integrate a DNA copy of their genome into the chromosomes of their hosts as part of their life cycle. Exogenous retroviruses (XRVs) spread infectiously and are usually inserted in the chromosomes of somatic cells [1]. However, endogenous retroviruses (ERVs) become permanently embedded in the host germline and are passed on genetically [2,3]. Multiple copies of different kinds of ERVs are found in the genomes of humans and other species [2,3]. This shows that these ERVs were able to spread within the genome via retrotransposition, although most copies may now be inactive. An ERV originates when an XRV inserts by chance into a germline cell rather than a somatic cell. It is then possible for the ERV to spread through the population via genetic inheritance, and to multiply within the genome via retrotransposition. Here, we investigate the probability that an ERV arising initially in a single individual will spread to fixation, meaning that all individuals will have at least one copy of the ERV. As the ERV arose from an XRV, there is likely to be an XRV infection in the population at the time the ERV is spreading, and we investigate here how the presence of the XRV affects the fixation probability of the ERV. Although ERVs appear to become inactive on long time scales (presumably due to loss-of-function mutations), the on-going spread of active ERVs can be observed when there has been a recent introduction of a new ERV, such as in the case of the koala retrovirus [4,5].

Horizontal transmission refers to transmission of an infectious virus to an unrelated individual. Vertical transmission refers to transmission to the offspring of an infected individual. However, the term vertical is ambiguous in the context of retroviruses because it does not distinguish between the infectious transmission of an XRV to offspring and the genetic transmission of an ERV to offspring. Many XRVs can be transmitted infectiously from mother to offspring. In mammals, infection can occur during pregnancy, childbirth, or breastfeeding. We refer to this as ‘maternal transmission’, as it comes specifically from the mother, while we use ‘genetic transmission’ to refer to the transmission of ERVs in the genome, which can occur from either parent. Maternal infection has been observed in the human immunodeficiency virus (HIV) [6,7], the human T-cell leukemia virus (HTLV) [8], the mouse mammary tumor virus (MMTV), the feline leukemia virus (FeLV), the bovine leukemia virus (BLV) [9], and in the koala retrovirus (KoRV) [4,5]. In birds, avian retroviruses can also be transmitted maternally via the egg [10].

Theoretical models suggest that maternal transmission favors an evolution of lower virulence, because a virus that severely harms its host reduces its own reproductive success [11,12,13]. In this paper, “virulence” refers to the reduction in fitness of the host (increased death rate) caused by the presence of the virus. This definition applies either to an XRV or an ERV. For an XRV, we suppose that transmission is related to virulence, because the level of activity of the virus in the host determines the rate of virion production as well as the strength of the negative effect on the host. For an ERV that is not transmissible, there may still be a negative effect on the host, but this may be much smaller. As an ERV does not need to produce large numbers of virions, it has the potential to evolve low virulence, as long as it can maintain the ability to transpose. We begin with a deterministic model for the evolution of virulence of an XRV and show that viruses that are well-adapted to maternal transmission evolve low virulence, and vice versa. We then consider a stochastic model for the spread of an ERV originating in a population infected by the XRV.

A single copy of an ERV gene that is unable to transpose in the genome will be eliminated by selection if it has any deleterious effect. If it is completely harmless to the host, it behaves as a neutral gene, and hence it has only a very small probability of fixation (1/2N for a diploid population of size N). However, if transposition occurs in a diploid sexually reproducing population, there can be a significant probability of fixation even if the ERV is deleterious to the host. The simplest case of a deleterious transposable element spreading at a single diploid locus was considered in [14]. Here, we estimate the fixation probability as a function of the transposition rate, the effect on host fitness, and the number of possible loci at which the ERV can insert.

Several types of interaction are possible between ERVs and XRVs which influence the fate of a newly-inserted ERV. An ERV can sometimes protect the host from subsequent infection by a related XRV. In this case, an inserted ERV provide a fitness benefit to the host, which increases its probability of spreading. Receptor interference refers to cases where endogenous proviruses protect against infections by exogenous retroviruses carrying related envelope glycoproteins [15,16]. For example, an envelope protein, Syncytin-1, derived from the Human Endogenous Retrovirus type W (HERV-W) bind to the ASCT2 receptor, preventing related XRVs from using the receptor for cell entry [17]. It was also found that endogenous forms of the feline leukemia virus (FeLV) play a role in protection against the exogenous form [18]. In addition to receptor interference, several other mechanisms are known by which ERVs provide protection against XRVs [19].

ERVs can also have negative consequences for the host. For example, expression of proteins from the endogenous Jaagsiekte sheep retrovirus induces immune tolerance in sheep, reducing their immune response to related exogenous viruses [20]. In some cases, ERVs can be reactivated by XRVs, leading to the production of recombinant viruses that may be more infectious or more virulent [21,22].

In this paper, our central aim is to investigate how likely it is that a newly-inserted ERV will spread to fixation. We consider the three following cases: an ERV spreading in an uninfected population; an ERV in a population infected by XRV where the ERV does not protect against XRV infection; and an ERV in a population infected by XRV where the ERV provides complete resistance to infection by the XRV.

## 2. Methods

### 2.1. Deterministic Model for Exogenous Viruses

We first consider the evolution of virulence of an exogenous virus. We consider a population with $N_{0}$ uninfected individuals and $N_{X}$ individuals infected by an XRV. The total population is $N_{tot}=N_{0}+N_{X}$, and the carrying capacity is K. The birth and death rates are $b_{0}$ and $v_{0}$ for uninfected individuals and $b_{X}$ and $v_{X}$ for infected individuals. The rate of production of the virus in the host cells is denoted by x. It is assumed that the virus is transmitted horizontally at a rate proportional to x. The rate of infection of an uninfected host is $\alpha xN_{X}/K$, where α is a contact rate between the hosts and $N_{X}/K$ is the frequency of the infected hosts. The virus is also transmitted maternally. The offspring of an infected mother is infected with the probability m and uninfected with the probability 1−m. The virus reduces the host fitness by increasing the death rate, such that $v_{X}=v_{0}e^{x}$. We suppose there is no effect on the birth rate, so $b_{X}=b_{0}$. This model is based on that of [11,12]. The ODEs are as follows:

(1) $$\frac{dN_{0}}{dt}=b_{0}N_{0}\left(1-\frac{N_{tot}}{K}\right)+b_{X}\left(1-m\right)N_{X}\left(1-\frac{N_{tot}}{K}\right)-v_{0}N_{0}-\frac{\alpha xN_{0}N_{X}}{K},$$

(2) $$\frac{dN_{X}}{dt}=b_{X}mN_{X}\left(1-\frac{N_{tot}}{K}\right)-v_{X}N_{X}+\frac{\alpha xN_{0}N_{X}}{K}.$$

The density of individuals in an uninfected population is $p_{uninf}=\frac{N_{0}}{K}=1-\frac{v_{0}}{b_{0}}$. When the infection is at low frequency, $\frac{dN_{X}}{dt}\approx \lambda N_{X}$, where $\lambda =b_{X}m\left(1-p_{uninf}\right)-v_{X}+\alpha xp_{uninf}$, the virus will spread when rare if *λ* > 0. This condition can be written as R>1, where the reproductive ratio R is defined as the mean number of additional infected hosts produced by a single infected host in its lifetime. In this case,

(3) $$R=\frac{b_{X}m}{v_{X}}\left(1-p_{uninf}\right)+\frac{\alpha xp_{uninf}}{v_{X}}.$$

Equations (1) and (2) can be generalized to consider multiple competing strains *i* with populations $N_{i}$, virus production rates $x_{i}$, maternal transmission rates $m_{i}$, and death rates $v_{i}=v_{0}e^{x_{i}}$. We assume the birth rate is $b_{0}$ for all strains. The total number of infected individuals is $N_{inf}=\sum_{i}N_{i}$, and the total number of individuals, including both infected and uninfected, is $N_{tot}=N_{0}+N_{inf}$. The ODEs are as follows:

(4) $$\frac{dN_{i}}{dt}=b_{0}m_{i}N_{i}\left(1-\frac{N_{tot}}{K}\right)-v_{i}N_{i}+\frac{\alpha x_{i}N_{0}N_{i}}{K},$$

(5) $$\frac{dN_{0}}{dt}=(b_{0}N_{0}+\sum_{i}b_{0}\left(1-m_{i}\right))\left(1-\frac{N_{tot}}{K}\right)-v_{0}N_{0}-\frac{\alpha N_{0}}{K}\sum_{i}x_{i}N_{i}.$$

These equations assume that, once a host is infected by one strain, it cannot be infected by another strain, and that once infected, it never recovers. New uninfected individuals arise from birth and not from recovery. If there is no maternal transmission (m=0), the reproductive ratio for strain *i* is $R_{i}=\alpha x_{i}p_{uninf}/\left(v_{0}e^{x_{i}}\right)$. The maximum R therefore occurs when x=1. If there are multiple strains with different $x_{i}$, but $m_{i}=0$ for all strains, the virus evolves toward the strain with the maximum R. Hence, if there is only horizontal transmission, the optimal virus has x=1. The parameter x represents the level of virus production in the host somatic cells, and we have assumed the infection rate is directly proportional to x. It is necessary for the death rate to increase faster than linearly with x, because if $v_{i}$ were simply proportional to $x_{i}$, there would be no optimal value of x, and the virulence would increase indefinitely. The relative death rate of an individual infected by the optimal strain and an uninfected individual is $e^{1}=2.718$. Thus, the horizontally transmitted virus has a large deleterious effect on the fitness of the host.

If maternal transmission also occurs (m>0), the virus is expected to evolve to a state with a lower virulence [11,12,13]. In this case, it is not true that the parasite evolves towards the strain which maximizes R. We determined the optimal parasite parameters numerically by considering the competition between multiple strains of the parasite. We assumed that the maternal infection rate is dependent on the viral production rate, $m_{i}=1-e^{-Mx_{i}}$. The parameter M determines the ease with which maternal transmission can occur. Strains with $x_{i}\gg 1/M$ have close to 100% maternal transmission. High M means maternal transmission is easy, so even strains with low x have high m. Low M means maternal transmission is difficult, so only strains with high x have a significant chance of maternal transmission.

The above equations do not explicitly distinguish between males and females. If we assume that the birth rate is limited by the reproductive capacity of the female, and not by the time taken to find a mate, then the birthrate should be proportional to the number of females ($N_{0}/2)$. We could write the birth terms as proportional to $N_{0}/2$ instead of $N_{0}$. However, if we redefine the birth rate per female as $2b_{0}$, these factors of 2 cancel out. The model for a sexual species with birthrate $2b_{0}$ per female and maternal (but not paternal) transmission is the same as the model for an asexual species with birthrate $b_{0}$ for all individual and vertical transmission from all individuals.

### 2.2. Stochastic Model for Endogenous Viruses

When considering endogenous viruses, we use a stochastic model in which there is a single type of XRV infection in the population at the same time as the transmission of the ERV genes. Each individual has a diploid genome with L loci at which insertion of the ERV gene is possible. Alleles at these loci are denoted by 0 or E for the absence or presence of an inserted ERV at this site. The number of E genes in one individual is between 0 and 2L. Individuals in the population consist of four types: 0, X, E, and EX. Type 0 individuals have no XRV infection and no E genes. Type X individuals are infected by the XRV but have no E genes. Type E individuals have at least one E gene and are uninfected by the XRV. Type EX individuals have at least one E gene and are also infected by the XRV. The numbers of individuals of each type are denoted by $N_{0},\;N_{X},\;N_{E},\;N_{EX}$. Each individual has a sex, which is male or female with equal probability. The numbers of males and females are $N_{M}$ and $N_{F}$. The total number of individuals is $N_{tot}=N_{0}+N_{X}+N_{E}+N_{EX}=N_{M}+N_{F}$.

The XRV is defined by the parameters x and m. We choose pairs of x and m that correspond to pairs selected by evolution in the deterministic model for XRVs, i.e., we assume the virulence of the XRV has evolved to an optimal state prior to the origin of the ERV.

In the early stages of ERV spread there might be a type of ERV that is both infectious and transmitted in the genome. Once inserted, however, an ERV does not need to retain the ability to transmit infectiously, because it can still spread in the genome, and it is likely that the ERV will soon lose the ability to transmit infectiously. For simplicity, we assume the ERV immediately becomes non-infectious after insertion. E genes are transmitted genetically, but there is no horizontal or maternal transmission of the ERV. The death rate of uninfected individuals is $v_{0}$. The death rate of type X individuals is $v_{X}=v_{0}e^{x}$, as before. The death rate of type E individuals is $v_{E}=v_{0}e^{y}$, where y determines the strength of the deleterious effect of the endogenous strain. ERVs are expected to be less harmful to their hosts than XRVs, so we expect y<x in most cases. The death rate of type EX individuals is assumed to be determined by the more virulent of the two viral entities, i.e., $v_{EX}=\max\left(v_{X},v_{E}\right)$. The rate of infection of an uninfected individual by the XRV is $\alpha x\left(N_{X}+N_{EX}\right)/K$, assuming that type X and type EX individuals are equally infectious. The endogenous virus may provide some degree of resistance to infection by the XRV. The rate of infection of a type E individual by the XRV is $\beta x\left(N_{X}+N_{EX}\right)/K$. If β=0, the ERV provides complete resistance to infection by the XRV, and type EX individuals do not arise. If β=α, the ERV provides no protection agains the XRV and the rate of infection of an individual is independent of whether it possesses the E genes.

The carrying capacity of the host population is K. Arrays of size K are defined at the beginning of the program to store variables of each individual. The initial frequencies of the uninfected and individuals are $p_{0}=N_{0}/K$ and $p_{X}=N_{X}/K$, which are obtained from the stationary values of the deterministic model (Equations (1) and (2)). Each element of the array is initiated with an uninfected individual with the probability $p_{0}$, or an infected individual with the probability $p_{1}$, or it remains empty (no living individual) with the probability $1-p_{0}-p_{1}$. Each living individual is initiated with a genome of entirely 0 alleles. After this, a single individual is added with one E allele. The program then moves forward in time using the Gillespie algorithm [23], which is a common way of dealing with stochastic models.

Birth events occur at the rate $2b_{0}(1-\frac{N_{tot}}{K})$ for every female. If a female gives birth, one random male is chosen as the father. An offspring is created with genes inherited from both parents, using the standard Mendelian inheritance for L unlinked loci. The offspring is placed in one of the empty elements of the population array. If the offspring inhertits no E genes and the mother is infected by the XRV (either type X or type EX), the offspring may be maternally infected with the probability m. If the offspring inherits at least one E gene and the mother is infected, maternal transmission of the XRV occurs with the probability mβ/α. Thus, if β=0 the ERV provides protection to maternal infection as well as to horizontal infection. The sex of the offspring is chosen as male or female randomly.

Death events occur at the rate $v_{0},v_{X},v_{E}$, or $v_{EX}$, according to the type of individual. If a death occurs, one random individual of the appropriate type is eliminated. Infections occur at the rate $\alpha x\left(N_{X}+N_{EX}\right)/K$ for an individual with no E genes, and at the rate $\beta x\left(N_{X}+N_{EX}\right)/K$ for an individual with at least one E gene. If an infection occurs, one random individual is changed from type 0 to type X, or from type E to type EX.

Transposition events occur at the rate r per gene. Insertions can only occur at sites that are not already occupied by an E gene. An individual having l ERV genes has a transposition rate $rl(1-\frac{l}{2L})$, where the factor in brackets is the fraction of sites that are not yet occupied. If a transposition occurs, a random 0 allele is selected from the genome of this individual and replaced by an E allele.

As is usual with the Gillespie algorithm, the total rate of events $R_{tot}$ is calculated at each time step. Then, one possible event is chosen randomly with a probability proportional to its rate. The elapsed time t is augmented by δt, which is chosen from an exponential probability distribution $P\left(\delta t\right)=R_{tot}\exp\left(-R_{tot}\delta t\right)$. The population is then changed due to the occurrence of the chosen event.

## 3. Results

### 3.1. Evolution of Virulence in an XRV with Maternal Transmission

Using the deterministic model for the XRVs described in the Section 2, we consider 1100 strains of XRVs, with strain *i* having $x_{i}=0.001i$, which gives values from 0.001 to 1.1. The maternal transmission rates are $m_{i}=1-e^{-Mx_{i}}$. The uninfected birth and death rates are $b_{0}=1$, and $v_{0}=0.2$; hence, the density of individuals in an uninfected population is $p_{uninf}=0.8$. The carrying capacity is nominally $K=10^{6}$, but the value of K is unimportant because population sizes scale in proportion to K. We begin the simulation with $N_{0}=p_{uninf}K$ uninfected individuals and $N_{i}=1$ for all strains. We follow the population sizes forward in time using the standard fourth-order Runge–Kutta method. The mean values of x and m at any point in time are $\frac{1}{N_{inf}}\sum_{i}x_{i}N_{i}$ and $\frac{1}{N_{inf}}\sum_{i}m_{i}N_{i}$. In the long-time limit, the mean values converge to steady-state values, which we take as the estimate of the optimal virus that will be selected over time.

**Figure 1:**
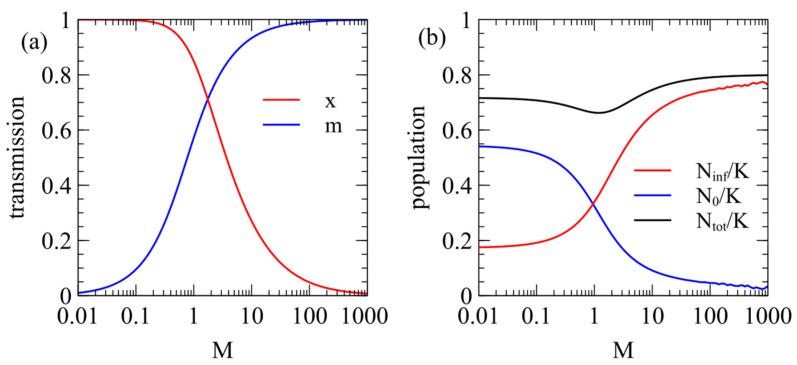
(**a**) Stationary-state parameters x and m as a function of M, the ease of maternal transmission. (**b**) Steady-state population sizes of infected and uninfected individuals.

Figure 1a shows the steady-state values of x  and m as a function of M with the contact rate fixed at α=1. When M≪1, there is very little maternal infection, m≈0, and the virulence is high, x≈1. For a larger M, m increases and x decreases. When M≫1, m≈1 and x≈0. Figure 1b shows the steady-state populations of infected and uninfected individuals. The fraction of infected individuals increases with M. For a low M, the virus is virulent, and a fairly small fraction is infected. For a very high M, almost the whole population is infected, but the virus has very little effect on the population.

**Figure 2:**
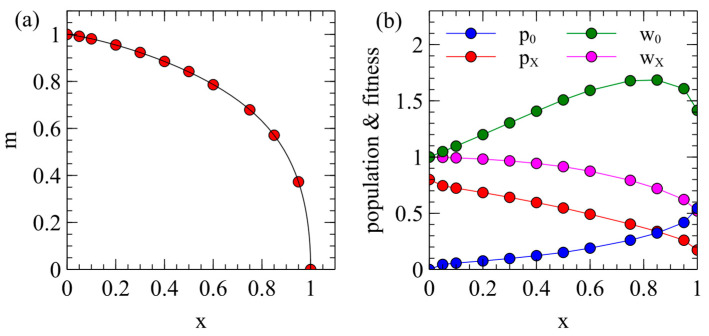
(**a**) Locus of points $\left(x,m\right)$ as M is varied, showing steady states of virulence and maternal transmission that are selected by evolution and competition between viral strains. Red points show cases that are used as examples in (**b**) and in later figures. (**b**) Populations of uninfected and infected individuals, $p_{0}=N_{0}/K$, and $p_{X}=N_{X}/K$, and relative fitness values $w_{0}$ and $w_{X}$ of uninfected and infected individuals, using the example cases from (**a**).

The steady-state x and m values from Figure 1a can also be plotted as in Figure 2a, which shows how the maternal transmission rate is expected to coevolve with virulence. Different viruses are expected to evolve to some point on this curve which will depend on the extent that maternal transmission is possible in that virus (i.e., on the M parameter).

Example points $\left(x,m\right)$ are marked on Figure 2a. For each of these points, we determined the populations of uninfected and infected individuals $p_{0}=N_{0}/K$, and $p_{X}=N_{X}/K$, when there is a single virus strain with the corresponding x and m. This is shown in Figure 2b. These values are used as starting conditions when we consider the introduction of an ERV later in this paper. The mean death rate of an individual in this population is $v_{mean}=\left(v_{0}p_{0}+v_{X}p_{X}\right)/\left(p_{0}+p_{X}\right)$. Fitness is inversely proportional to the death rate, so the relative fitness of an uninfected individual is $w_{0}=v_{mean}/v_{0}$ and the relative fitness of an infected individual is $w_{X}=v_{mean}/v_{X}$. These fitness values are shown in Figure 2b. The value of $w_{X}$ decreases steadily with x because $v_{X}$ increases with x; however, $w_{0}$ has a maximum around x=0.7–0.8. Although $v_{X}/v_{0}$ would be the maximum at x=1, $v_{mean}/v_{0}$ is the maximum for smaller x because a larger fraction of the population is infected when x is smaller. This gives a maximum in the $w_{0}$ curve which is relevant later when we consider the spread of ERVs.

**Figure 3:**
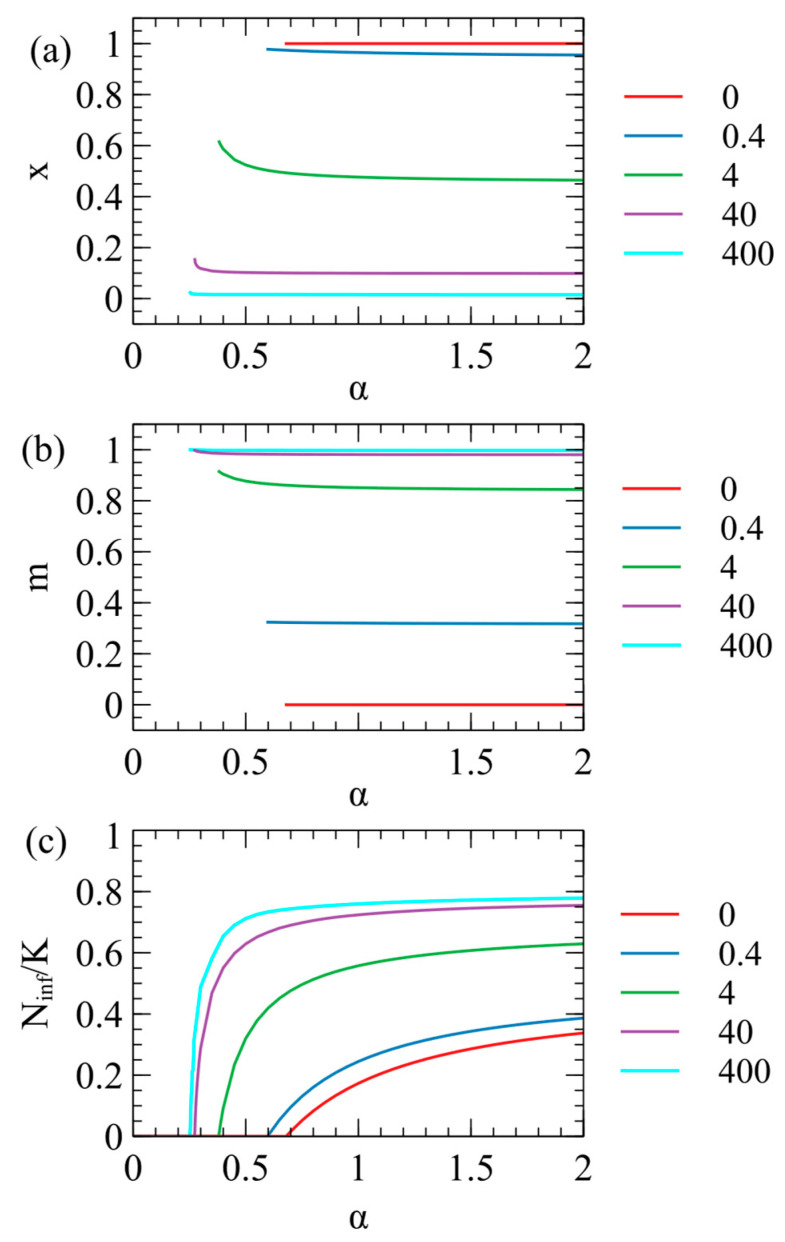
Steady-state parameters for XRVs as a function of contact rate α: (**a**) virulence x; (**b**) maternal transmission rate m; (**c**) number of infected individuals $N_{inf}/K$. Curves show cases with M=0, 0.4, 4, 40, 400.

In Figure 1 we considered a fixed value of the contact rate α=1. However, the optimal parameters for the virus also depend on α, as shown in Figure 3. We suppose that α depends on the behaviour of the host population (rate of close contacts between the hosts) and is not controlled by the virus; hence, virus evolution depends on α but α does not change when the virus evolves. We considered the evolution of the optimal virus as a function of α for several fixed values of M. For each value of α, we began with 1100 strategies, as above, and determined the steady-state values of x and m in the long-time limit. The virus survives for α greater than a critical value $\alpha_{c}$, which is different for each value of M. The optimal x and m values are only defined when the virus survives; therefore, they are only plotted for $\alpha >\alpha_{c}$ in Figure 3a,b. In Figure 3c, we show the number of infected individuals $N_{inf}/K$, which is zero for $\alpha <\alpha_{c}$, and increases with α for $\alpha \geq \alpha_{c}$. The critical contact rate is $\alpha_{c}=0.68$ for M=0 and decreases as M increases.

When M=0, there is no maternal transmission (m=0) and x=1 for all values of $\alpha >\alpha_{c}$. Larger M values give larger m and smaller x, i.e., the presence of maternal infection leads to the evolution of lower virulence, as expected. For very large M (e.g., M=400 in Figure 3), m is very close to 1, and x is very close to 0, i.e., the virus is almost harmless to the host and relies almost exclusively on maternal transmission. It is not possible to have x strictly equal to 0, however, since a parasite with only vertical transmission can never survive if there is even a tiny deleterious effect on the host.

As α decreases towards $\alpha_{c}$, the optimal x inreases slightly and there is a corresponding increase in m. This is most easily seen for M=4 in Figure 3a,b, but also occurs in the other curves. As α decreases, there are fewer opportunities for contact between hosts, therefore strains with higher infection rates per contact (higher x) are selected. This means that strains with low virulence and a high maternal transmission rate are most likely to evolve when the opprotunity for horizontal transmission is high (high α), as has been noted previously [13]. However, Figure 3 shows that for α≥1 there is little change of x and m with α, so we we fix α=1 for all the subsequent examples.

### 3.2. Spread of an ERV in an Uninfected Population

Using the stochastic model for ERVs described in the Section 2, we first consider a case where the ERV spreads in a population of uninfected individuals. The carrying capacity is K=1000, therefore the number of ininfected individuals is initially $N_{0}\approx Kp_{uninf}=800$. At time zero, one of these individuals is turned into a type E individual, and a single copy of the ERV is added to its genome. The number of individuals with at least one ERV copy, $N_{E}$, is followed over time. The simulation is continued until either the ERV is eliminated ($N_{E}=0$) or the ERV spreads to fixation, so there are no remaining individuals without the ERV ($N_{0}=0$). We ran $N_{repeats}$ simulations for each parameter set and measured $p_{fix}$, the fraction of runs that reaches fixation. In most cases, $N_{repeats}=10^{4}$. We denote fixation as the point when every individual has at least one ERV copy. This does not mean that the ERV is inserted at every locus, in which case every individual would have 2L copies. However, once every individual has at least one copy, the ERV continues to multiply slowly by transposition until every individual has 2L copies. When measuring the fixation probability, we stopped the simulations at the point when every individual has at least one copy, because this saves considerable computer time.

An ERV in an uninfected population is equivalent to a transposable element that spreads by transposition and genetic inheritance. This case is somewhat artificial for an ERV, because we presume the ERV arose by the insertion of an XRV, so the XRV must also be present. However, we begin by considering the spread of the ERV in the absence of the XRV because this provides a baseline with which to compare other cases.

**Figure 4:**
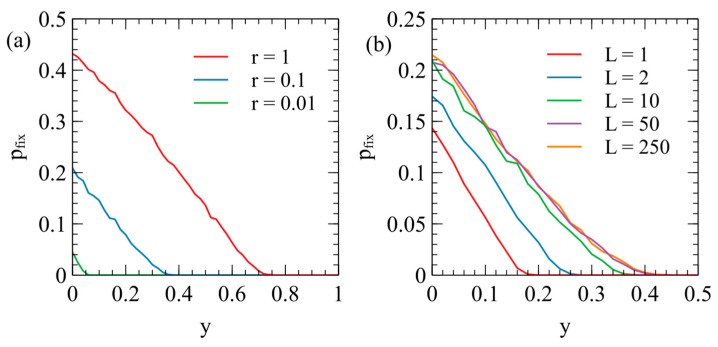
(**a**) The probability of fixation of an ERV in an uninfected population as a function of the deleterious effect y of the ERV shown for three values of the transposition rate r. The number of loci is L=10 and the number of repeat runs is $N_{repeats}=10^{4}$. (**b**) The effect of varying the number of loci L from 1 to 250. In all cases, r=0.1.

As described in the Section 2, the death rate of an individual with at least one ERV copy is $v_{E}=v_{0}e^{y}$, where y is the deleterious effect of the ERV. Figure 4a shows $p_{fix}$ as a function of y for several values of the transposition rate r. The fixation probability is highest for the smallest y, because low y ERVs have the least deleterious effect on host reproduction. For a high transposition rate, r=1, there is a measureable chance of fixation for y as high as 0.7 (i.e., at least 1 out of the 10,000 repeat runs reached fixation). For smaller transposition rates, only lower y values have a measurable chance of fixation.

It is also possible to consider the limit of r=0, in which case the ERV cannot transpose, but still has some chance of spreading to fixation at the single locus in which it was introduced. If r=0 and y=0, the ERV is a neutral gene at a single locus. The probability of fixation of a neutral gene beginning with a single copy is $1/2N_{tot}$. In this case, $N_{tot}=800$ and $p_{fix}=6.25\times 10^{-4}$. We ran this data point for $N_{repeats}=10^{5}$, and found 60 cases of fixation, which is consistent with the neutral theory within statistical error. We also ran the case of r=0 for the full range of y with $N_{repeats}=10^{4}$, and found the fixation probability was too small to measure for non-zero values of y. As shown in Figure 4a, the fixation probability for ERVs with a non-zero transposition rate is very much higher than expected for a single neutral gene without transposition ($p_{fix}=6.25\times 10^{-4}$). ERVs with a significant deleterious effect have high fixation probabilities if r is high enough, whereas a single deleterious gene without transposition would have a negligible fixation probability on the scale of Figure 4a.

The fixation probability also depends on the number of available loci L into which the ERV can insert. We assumed that, if there is currently one ERV copy in a genome, the rate of transposition is proportional to 1−1/2L, the fraction of sites that are not yet occupied by an ERV. This factor gives a significant reduction in the multiplication rate of the ERV if L is small. The examples in Figure 4a were calculated with L=10. Figure 4b shows cases of L=1, 2, 10, 50, and 250, with r=0.1 in each case. For L = 1 and 2, $p_{fix}$ is significantly lower than for L=10. For L = 50 and 250, $p_{fix}$ is only slightly higher than for L=10 because the effect is of the order 1/2L. For the rest of this paper, we consider only L=10, because increasing L makes only a small difference to the result and increases the required simulation time by a large amount.

### 3.3. Spread of an ERV in the Presence of an XRV

We now consider the case where the ERV arises in a population infected by an XRV. The XRV is assumed to have evolved to parameters x and m that are optimal parameters from the model for the evolution of virulence of the XRVs. We fix the contact rate at α=1. We use the x and m values marked as points in Figure 2a as parameters for the XRV. The stochastic model begins with numbers of uninfected and infected individuals $N_{0}$ and $N_{X}$ for the corresponding x and m (shown in Figure 2b). At time zero, one type X individual is changed to a type EX individual, and one copy of the ERV is added to its genome. We follow $N_{0},\;N_{X},\;N_{E},\;N_{EX}$ as a function of time until either the ERV is eliminated ($N_{E}+N_{EX}=0$) or reaches fixation ($N_{0}+N_{X}=0$). We define g as the mean fraction of loci occupied by the ERV, which is the mean number of ERV copies per individual divided by 2L. The parameters are as previously established, as follows: $K=1000,\;v_{0}=0.2,\;b_{0}=1,\;L=10$.

**Figure 5:**
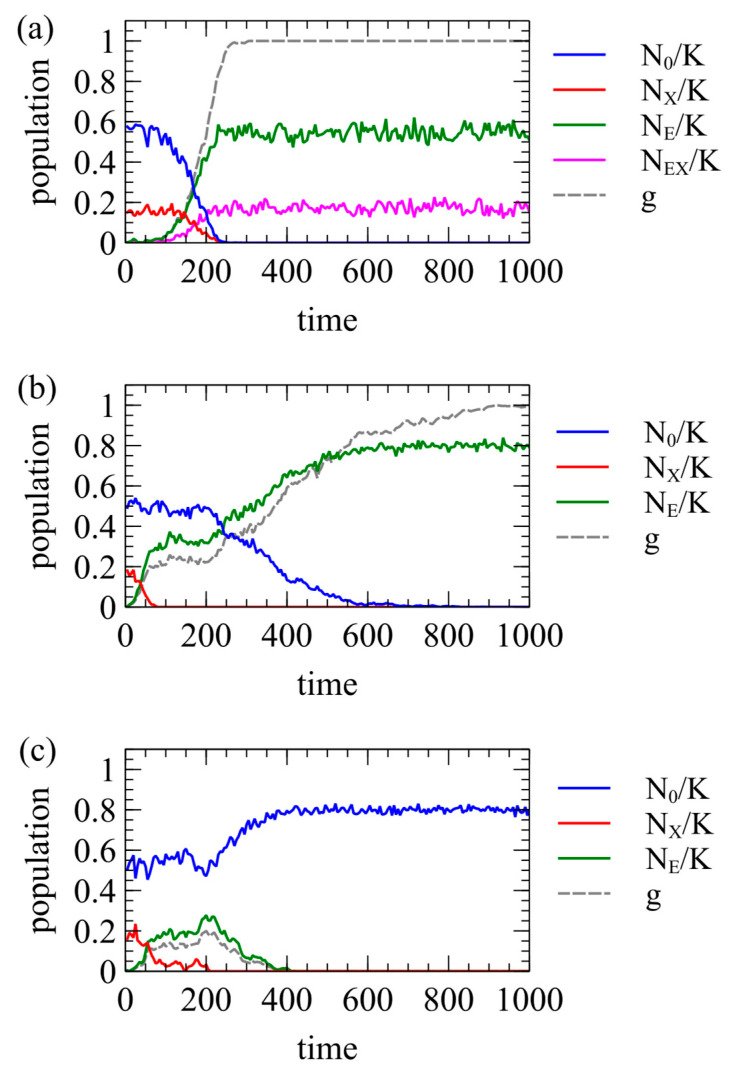
Example runs showing spread of an ERV in the presence of an XRV. (**a**) x=1, y=0, r=0.1, β=1. The ERV spreads to fixation. The XRV is still present. (**b**) x=1, y=0, r=0.01, β=0. The ERV spreads to fixation. The XRV is eliminated. (**c**) x=1, y=0.1, r=0.1, β=1. The ERV eliminates the XRV, and then is itself eliminated. Both XRV and ERV are cleared from the population.

Figure 5 shows three examples of single simulation runs. In all three cases, the XRV has x=1, m=0. In Figure 5a, y=0, r=0.1, and β=1, meaning the ERV is completely harmless to the host and offers no resistance to the XRV. In this case, the ERV spreads to fixation quite quickly. All individuals have at least one ERV at time 290, and g=1 shortly afterwards, at time 305. In this case, individuals with the ERV can still be infected by the XRV. Therefore, individuals of type EX and type E remain at the end of the run. In Figure 5b, y=0, r=0.01, and β=1, meaning that the ERV offers complete resistance to the XRV. The ERV spreads more slowly because r is lower, but fixation still occurs. All individuals have at least one ERV at time 895, and g=1 at time 1010. In this case, the ERV provides complete resistance to the XRV. The first insertion of the ERV occurs in an infected individual, so one type EX individual is created at time zero, but the offspring inheriting the ERV cannot be infected by the XRV. Therefore, there are no further type EX individuals after the original one dies. At the end of the run, all individuals are type E. For both Figure 5a,b, we have chosen runs that reach fixation. However, the outcome is stochastic, and there are also many other runs for the same parameters in which the ERV is eliminated almost immediately.

Figure 5c shows what we call “clearance”. In this case, y=0.1, r=0.01, and β=1. As y<x, type E individuals have higher fitness than type X individuals. Therefore, type E individuals increase initially, and type X individuals decrease. After the disappearance of the XRV at time 210, type E individuals have a lower fitness than uninfected individuals because y>0. Therefore, type E individuals are selected against, and the ERV is eliminated at time 425. Thus, the introduction of the ERV leads to clearance of the XRV from the population, even when it does not itself reach fixation.

**Figure 6:**
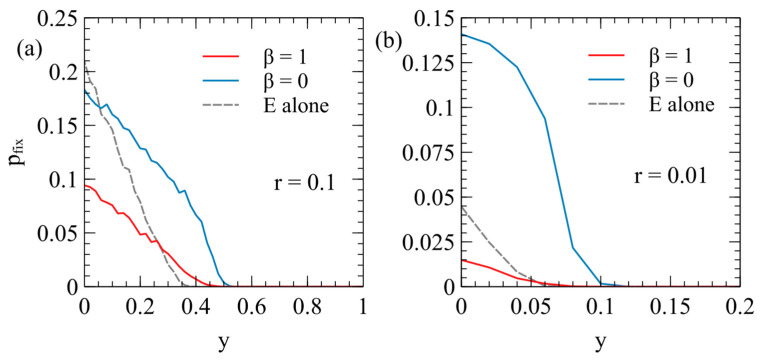
The probability of fixation of an ERV with the deleterious effect y in a population originally infected by an XRV with x=1 and m=0: (**a**) r=0.1, (**b**) r=0.01. In each case, the cases β=1 and β=0 are compared with the case where the ERV spreads alone in an uninfected population.

Figure 6 shows the fixation probability of the ERV as a function of its deleterious effect, y, in a population originally infected with a virulent XRV, with x=1 and m=0. Two different transposition rates are shown in Figure 6a,b. In each case, the fixation rates are measured separately for β=1 and β=0, with $N_{repeats}=10^{4}$ for each data point. These are compared with the fixation probability when the ERV spreads alone in an uninfected population (dashed curves are the same as Figure 4a).

There are two principle factors that cause the fixation probabilities to be different in the presence of the XRV from when the ERV is alone. Firstly, chance events immediately after insertion of the first ERV copy have a significant influence on $p_{fix}$. For example, if the first individual with a single ERV copy dies before reproduction, then the ERV disapears immediately. If the first ERV is inserted in an infected individual, this individual is type EX, and has the death rate $v_{EX}=v_{0}e^{x}$ (because the death rate is determined by the XRV when x>y); whereas, if the ERV is alone, the first ERV is in a type E individual with the death rate $v_{E}=v_{0}e^{y}$. Thus, the ERV is more likely to be eliminated immediately after insertion if it arises in a population with the XRV present. This effect will reduce the fixation probability in the presence of the XRV relative to that in populations without the XRV. Secondly, however, if the first individual successfully passes on the ERV gene, then a new type E individual is created, which has a lower death rate than individuals with the XRV infection. The type E individual is then competing with a mixture of type X and type 0 individuals and may have a higher fitness than the average of the X and 0 types. This effect tends to increase $p_{fix}$ relative to the case of the uninfected population. In an uninfected population, E types are competing against only uninfected individuals, and they always are less fit than the uninfected individuals. The advantage of ERV in the presence of the XRV is particularly important when β=0, because in this case, all individuals inheriting the ERV gene are resistant to infection by the XRV. When β=1, there are both type E and type EX individuals containing ERV genes, so the fitness advantage to the E genes is less. This explains why $p_{fix}$ is larger for β=0 than it is for β=1. A comparison of Figure 6a,b shows that the second effect is larger when the transposition rate r is smaller. For the case r=0.01, $p_{fix}$ with β=0 is very much larger than when β=0 or when the ERV is alone.

**Figure 7:**
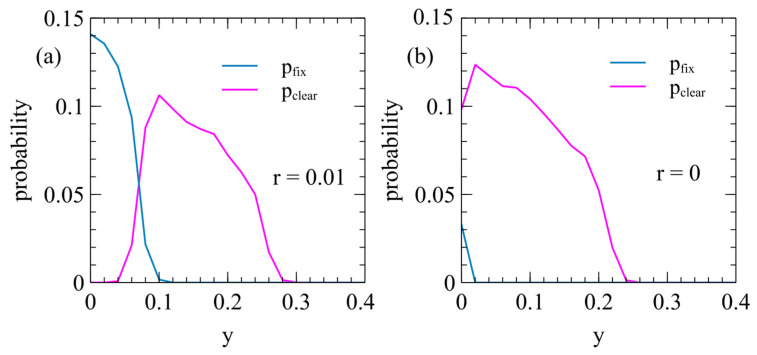
Spread of an ERV in a population originally infected by an XRV with x=1 and m=0. The probabilities of fixation and clearance are shown as a function of the virulence y of the ERV: (**a**) r=0.01, (**b**) r=0.

Figure 7 shows the probabilities of clearance and fixation as a function of y in the case where the ERV with the deleterious effect y and β=0 spreads in a population with an XRV with the deleterious effect x=1. Clearance happens frequently when the transposition rate is small (as in Figure 7a, with r=0.01). Clearance occurs when y is non-zero, but is substantially less than x, so the ERV has a high relative fitness initially while the XRV is still present, but has a low relative fitness compared to uninfected individuals. Clearance also occurs frequently in the limiting case when the ERV cannot transpose (r=0 as in Figure 7b). In this case, there is a wide range of y where clearance occurs, but fixation occurs only when y=0.

**Figure 8:**
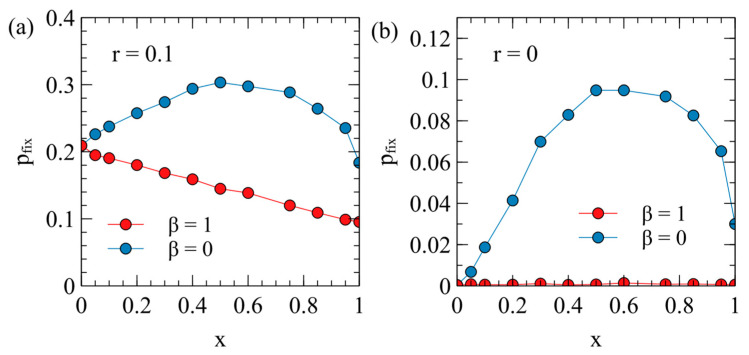
The fixation probability of a harmless ERV (y=0) in a population with an XRV of virulence x and a corresponding maternal transmission rate m selected by evolution (as in Figure 2a). (**a**) when r=0.1, (**b**) when r=0.

The case that seems most relevant for real endogenous viruses is when the ERV is almost harmless to the host. We therefore consider the fixation probability for an ERV with y=0 as a function of the virulence x of the XRV that is present. For each data point in Figure 8, a value of x is chosen together with the corresponding maternal transmission rate m from the points on Figure 2a.

Figure 8a has the transposition rate r=0.1. When x=1, the fixation probability with β=0 is about twice that with β=1 (this case is the same as the point y=0 in Figure 6a). For β=1, $p_{fix}$ decreases steadily with x, while for β=0, $p_{fix}$ is largest in the range x = 0.5–0.8. When x=0, the XRV has no effect on fitness, so the fixation probabilities for β=0 and β=1 are both equal to the fixation probability in absence of an XRV. The shape of the curves in Figure 8a can be explained by reference to Figure 2b, where we calculated the relative fitness values $w_{0}$ and $w_{X}$ for uninfected and infected individuals. When β=0, type E individuals have the death rate $v_{0}$. The fitness of these individuals relative to the average population shortly after introduction of the ERV is $w_{0}$; therefore, the fixation probability for β=0 in Figure 8a follows a similar shape to $w_{0}$ in Figure 2b. For β=1, most of the individuals with ERV genes are type EX and have the death rate $v_{0}e^{x}$; therefore, the fixation probability for β=1 in Figure 8a follows a similar shape to $w_{X}$ in Figure 2b.

Figure 8b considers the limit of a zero transposition rate. In this case, $p_{fix}$ is very small for all values of x when β=1, but is still quite large when β=0. A single copy of a non-transposable ERV behaves like a resistance gene whose advantage is proportional to the relative fitness of an uninfected individual with to respect to the mean population fitness. The fixation probability for β=0 therefore has a similar trend to the $w_{0}$ curve in Figure 2b.

**Figure 9:**
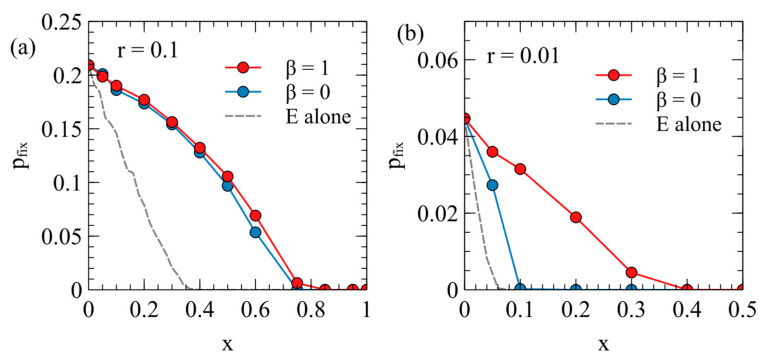
The probability of fixation of an ERV in a population infected by an XRV in the case where both have equal virulence, y=x, in comparison to the probability of fixation when the ERV spreads alone: (**a**) r=0.1, (**b**) r=0.01.

If the ERV has y=0, then it must have changed in some way from the XRV in order to be less harmful to the host. Mutations which limit the harmful effect of an ERV are likely to evolve because this increases the spread rate of the ERV. However, such mutations may not have occurred in the early stages after the introduction of the ERV. Therefore, in Figure 9 we consider a case where the virulence of the ERV is the same as the virulence of the XRV from which it originates, y=x. Even so, the ERV and XRV are different in this case, because the XRV has horizontal and maternal transmission, whereas the ERV is transmitted genetically.

Figure 9 shows the fixation probabilities as a function of x in the case y=x. The fixation probabilities for both β=1 and β=0 are higher than the case when the ERV is alone. This is because type E individuals are competing against the average of the rest of the population, and many of these individuals are type X. By contrast, when the ERV spreads alone, all the competing individuals are type 0. Figure 9b shows that, for smaller transposition rates, $p_{fix}$ is lower for β=0 than for β=1, while the reverse was true in Figure 6 and Figure 8. In other words, when y=x, the ERV has a lower chance of fixation if it confers resistance the the XRV than if it does not. This can be understood by noting that, when β=0, as type E individuals increase, the fraction of individuals that are susceptible to the XRV decreases, and the XRV infection tends to be eliminated (clearing); so to reach fixation, the type E individuals have to compete against uninfected individuals. Whereas, when β=1, type E individuals are competing against mostly type X individuals, which have the same fitness as themselves in this case.

## 4. Discussion

The high incidence and diversity of ERVs in eukaryotic genomes [2,3] implies that ERVs spread to fixation quite frequently on the evolutionary time scale. In our model, the parameter y controls the effect of the ERV on the host death rate, which is $v_{0}e^{y}$. In the simplest situation, where the ERV is spreading in an uninfected population, individuals with the ERV are competing against uninfected individuals with the death rate $v_{0}$. Fixation of the ERV only occurs if the transposition rate r is high enough to overcome any deleterious effect of the virus. Figure 4 shows that, if r is high enough, ERVs with a significant negative effect on the host can spread with high probability, while for small r, only ERVs that are almost harmless to the host will spread. In our model, the mean lifetime of an uninfected individual is $1/v_{0}$, i.e., five time units when $v_{0}=0.2$; so, if r=1, there will be five transposition events during the lifetime of a single infected individual. The rate r=1 is probably unrealistically high. The situation with few new transpositions per lifetime ($r/v_{0}<1$) seems more realistic. This is consistent with the observation that many ERVs currently in genomes appear to be inactive. However, this does not imply that they were inactive and harmless during the period in which they were originally spreading in the genome.

Our model is intentionally simple and leaves out many complexities relevant for ERV spread in nature. One relevant factor is that, for a newly inserted ERV to spread, it must avoid defense systems in the host that limit its proliferation, such as APOBEC, TRIM5α, and PIWI [24,25,26]. These systems are part of what determines r for a given ERV. In our model, we interpret r as the transposition rate in the presence of any host defence systems. However, it is possible that mutations may occur in the ERV after its initial insertion. If the original ERV has a low r, variants with a higher r may be selected because they spread faster. One reason that a higher r might be possible would be an adaptation that allows the ERV to avoid host defence systems. Mutations might also create inactive variants that can no longer transpose. Changes in the transposition rate during the period of spread will significantly affect the fixation probability, and this would be an interesting factor to consider in a more complex model.

The ERV might also change in other ways during its period of spread. We assumed in our model that the ERV was immediately non-infectious. However, it may be that the initially inserted ERV retains its ability to transmit infectiously as well as being transmitted in the genome. The ability to assemble virions and be transmitted infectiously could then be lost by mutations occurring during the period of spread, resulting in a non-infectious ERV. Our model already shows that a non-infectious ERV can spread, so later mutations that caused loss of infectious transmission would not prevent the ERV spreading. We would expect that adding an intermediate stage of infectious ERV would not greatly change the results of our present model. However, if the initial ERV were still infectious, it would presumably have a large negative effect on its host, i.e., the y of the ERV would be the same as the x of the XRV. Subsequent loss of infectious ability might be due to a reduction of virus activity in the host, which would mean the non-infectious ERV would have a lower fitness effect y. Changes in y might also occur due to mutations, and we would expect variants with a lower y to be selected. However, the fitness effect of a non-infectious ERV on its host must depend to some extent on its transposition rate, so it may not be possible to evolve a very low y without also having very low r. All these factors would be interesting to consider in a more complex model. Although there are experimental studies of recent invasions of ERVs [4,5], the fitness effect of the ERV and the transposition rate do not seem to have been measured, and these may be difficult to measure in practice.

Our model has considered the possibility that an ERV provides a selective advantage to the host by giving resistance to the XRV. However, there are other independent reasons why the ERV might confer an advantage. For example, the Jaagsiekte sheep retrovirus (JSRV) plays a beneficial role in the development of the placenta [27,28,29]. In humans, the ERV-derived Syncytin gene plays a role in the placenta [30,31], while HERV-H helps to maintain pluripotent stem cells [32,33,34]. An ERV conferring an advantage by one of these other means would be able to spread as a beneficial gene, and its success would not depend on its ability to transpose or on the continuing presence of the XRV in the population. Advantageous effects, such as these, may require changes in the virus that take some time to arise; whereas, if the ERV confers an advantage due to receptor interference, this will apply immediately after insertion, as in the current model. If there is divergence between ERV and XRV during the period of spread, then receptor interference might cease to operate. A change in the XRV that prevents receptor interference would be beneficial to the XRV, while a change in the ERV that prevents receptor interference would be disadvantageous to the ERV.

Our model shows that, if the ERV is spreading in a population already infected by the XRV, the fitness effect x of the XRV influences the probability of fixation of the ERV. Individuals with ERV genes are in competition with individuals without these genes, and the fitness of the individuals without the ERV is lower if some of them are infected with the XRV. This explains why the the fixation probability in the case when the ERV and XRV have equal virulence (y=x in Figure 9) is higher in the presence of the XRV than in absence. In the case where a virulent XRV is present and the ERV is less harmful (x=1, y≤x in Figure 6), the fixation probability may be either higher or lower than in the absence of the XRV. The advantage given to the ERV by the presence of the XRV is particularly important when the ERV prevents infection by the XRV (β=0 in our model). The advantage to an ERV with β=0 is very large when r is small (Figure 6b) and is important even in the limit r=0 (Figure 8b). A harmless, non-transposing ERV (y=0, r=0) has negligible chance of spread if it provides no protection against the XRV (β=1) but has a large fixation probability when it gives resistance to the XRV (β=0).

We considered maternal infection in this paper because many retroviruses have a significant probability of maternal infection. In the early stages of the HIV epidemic, when therapy was unavailable, it was estimated that between 13% and 43% of the offspring of infected mothers were infected [6]. In the case of HTLV-1 infected mothers, between 15% and 24% of the children were infected via breast-feeding [35]. A high frequency of maternal transmission of STLV-1 was observed in Japanese macaques [36], together with a high proportion (over 60%) of individuals that were infected. This is consistent with Figure 1, which shows that if M is high, the virus evolves high m and low x, and a high proportion of the population becomes infected. The macaque study [36] also shows the progressively increasing frequency of infected individuals with age and the progressively increasing viral load with age in infected individuals. This suggests that it would be interesting in future to study the effects of an age-structured population on the evolution of virulence and maternal transmission rate.

Maternal infection is relevant for retroviruses because they usually remain in the host for the whole lifetime, so there is a high chance that an infected mother will reproduce while infected. For other types of viruses where the host recovers after a short duration of infection, it is unlikely that a mother is infected at the time she gives birth, and maternal infection is less relevant. Given that infected mothers are likely to reproduce in the retrovirus case, to what extent the virus will grow depends on maternal transmission. Our model assumes that the probability of maternal transmission is a function of the virulence $m\left(x\right)=1-\exp\left(-Mx\right)$. Hence, it is only possible to have m close to 1 when x is high, and if x=0 then also m=0. The balance between m and x is determined by the parameter M, which we assume to be different for different viruses. Competition between viral strains selects an optimal combination of x and m lying on the curve in Figure 2a, hence the observed m may lie anywhere between 0 and 1. We have also considered variant models for the XRV virulence in which x and m evolve independently, but we find m either evolves to 0 or 1 in that case, which is inconsistent with the observation of intermediate m in the examples of the real viruses given above.

We observe that interactions between an ERV and an XRV often cause elimination of the XRV. If the ERV provides resistance to the XRV then the ability of the XRV to spread is diminished as the ERV increases in frequency, and the number of susceptible individuals eventually becomes too low to sustain the XRV. However, an unexpected result emerging from our study is that the XRV is frequently eliminated even without the ERV reaching fixation, and the result is that the introduction of an ERV causes the clearance of both XRV and ERV from the population.

Our model highlights several ways in which the interactions between an XRV and an ERV affect the likelihood of spread of the ERV. It has been kept deliberately simple, but it can serve as a basis for a more complex model in which ERV properties change due to mutations during the period of spread, and several competing forms of ERV exist simultaneously in the population.

## Data Availability

Programs used for study of the models in this paper are available at https://zenodo.org/records/15102539 (accessed on 25 March 2025).
